# *OsCSD2* and *OsCSD3* Enhance Seed Storability by Modulating Antioxidant Enzymes and Abscisic Acid in Rice

**DOI:** 10.3390/plants13020310

**Published:** 2024-01-20

**Authors:** Xiaohai Zheng, Zhiyang Yuan, Yuye Yu, Sibin Yu, Hanzi He

**Affiliations:** 1 College of Plant Science and Technology, Huazhong Agricultural University, Wuhan 430070, China; xiaohaizheng_3307@163.com (X.Z.); yuanzhiyang1989@163.com (Z.Y.); tripley2019@163.com (Y.Y.); ysb@mail.hzau.edu.cn (S.Y.); 2 National Key Laboratory of Crop Genetic Improvement, Huazhong Agricultural University, Wuhan 430070, China; 3 Beijing Bio Huaxing Gene Technology Co., Ltd., Beijing 102260, China

**Keywords:** seed storability, superoxidase, antioxidant capacity, phytohormone

## Abstract

Seed deterioration during storage poses a significant challenge to rice production, leading to a drastic decline in both edible quality and viability, thereby impacting overall crop yield. This study aimed to address this issue by further investigating candidate genes associated with two previously identified QTLs for seed storability through genome association analysis. Among the screened genes, two *superoxide dismutase* (*SOD*) genes, *OsCSD2* (*Copper/zinc Superoxide Dismutase 2*) and *OsCSD3*, were selected for further study. The generation of overexpression and CRISPR/Cas9 mutant transgenic lines revealed that *OsCSD2* and *OsCSD3* play a positive regulatory role in enhancing rice seed storability. Subsequent exploration of the physiological mechanisms demonstrated that overexpression lines exhibited lower relative electrical conductivity, indicative of reduced cell membrane damage, while knockout lines displayed the opposite trend. Furthermore, the overexpression lines of *OsCSD2* and *OsCSD3* showed significant increases not only in SOD but also in CAT and POD activities, highlighting an augmented antioxidant system in the transgenic seeds. Additionally, hormone profiling indicated that ABA contributed to the improved seed storability observed in these lines. In summary, these findings provide valuable insights into the regulatory mechanisms of *OsCSDs* in rice storability, with potential applications for mitigating grain loss and enhancing global food security.

## 1. Introduction

Rice is the most widely consumed food in the world. However, we also face the fact that a large amount of rice has to be stored for some time until usage, and seed deterioration during storage is a serious problem for rice production. In recent years, approximately 15 billion kilograms, equivalent to 3% of the average annual rice grain production, has been lost due to seed aging during storage [1]. Seed deterioration causes a decrease in edible quality as well as viability and consequently affects the preservation of germplasm resources and imposes enormous threats to crop production and food safety.

Seed storability, defined as the ability to remain alive during storage, is a critical trait for gene banks to conserve plant genetic resources during long-term storage. It is also a very important trait for farmers to ensure crop productivity, as it governs seed germination and seedling vigor for the next generation. In rice, through quantitative trait loci (QTL) analysis and association mapping approaches, more than 70 QTLs for seed storability have been identified under natural storage or artificial aging [2]. The pursuit of understanding seed longevity was initiated in 2002, marked by the identification of three QTLs—*qLG-2*, *qLG-4*, and *qLG-9* by accelerated aging treatment using 98 backcross inbred lines (BILs) derived from the *japonica* variety Nipponbare and the *indica* variety Kasalath [3]. Subsequent investigations revealed three seed storability QTLs—*qLS-9*, *qLS-11*, and *qLS-12*—using a doubled-haploid population. Xue, et al. [4] extended the understanding of seed storability by identifying three QTLs on chromosomes 1, 3, and 9 through Recombinant Inbred Lines (RILs) derived from the cross IR24/Asominori. During natural aging, Li, et al. [5] identified six QTLs affecting seed storability on chromosomes 2, 3, 4, 6, 9, and 11 in a BIL population of Koshihikari/Kasalath. Notably, a QTL on chromosome 9 emerged as a consistent locus across multiple genetic populations, affirming its stability in influencing seed storability [3,5,6]. In 2015, the research achieved a critical milestone as the QTL (*qLG-9*) was fine-mapped within a 30 kb interval with two genes (encoding trehalose-6-phosphate phosphatase (TPP) (Os09g0369400) and an unknown protein (Os09g0369500)) annotated in this region [7].

The regulation of seed deterioration involves a complex interplay between various endogenous genetic factors and external environmental cues. Endogenous factors encompass the balance of reactive oxygen species (ROS), hormonal levels, the integrity of nucleic acids and proteins, and the structure of the seed coat. External environmental stresses, including fluctuations in relative humidity, temperature, and oxygen partial pressure, significantly impact seed storability [8]. External stresses expose plants to heightened levels of ROS, leading to oxidative injury—a primary contributor to seed deterioration. The delicate equilibrium between ROS production and the antioxidant capacity of plants plays a pivotal role in determining seed storability [9]. Low concentrations of ROS play an important role in signal transduction in living organisms, enabling them to perceive the detrimental effects of environmental stresses in a timely manner and respond with defensive responses [10,11,12]. Conversely, elevated ROS concentrations lead to destructive oxidation of important cellular substances in seeds, including lipid peroxidation and degradation of polyunsaturated fatty acids. This process leads to the generation of harmful substances such as malondialdehyde (MDA), which attack macromolecular compounds, reducing the integrity of nucleic acids and proteins, disrupting seed structure and shortening seed longevity [13].

Plants have evolved a highly active antioxidant defense system to effectively counteract damage induced by oxidative stress, which is bifurcated into two components: non-enzymatic antioxidants and antioxidant enzymes. The main non-enzymatic antioxidants include phenolic compounds, alkaloids, non-protein amino acids, glutathione (GSH), ascorbic acid (AsA), vitamins, and β-carotene. Antioxidant enzymes primarily consist of superoxide dismutase (SOD), catalase (CAT), peroxidase (POD), ascorbate oxidase (APX), glutathione reductase (GR), etc. [14]. SOD plays a central role as the first line of defense against oxidative stress. According to the differences in metal cofactors bound to them, the *SOD* gene family is classified into four subfamilies—*Cu/Zn-SOD* (*CSD*), *Mn-SOD*, *Fe-SOD*, and *Ni-SOD*—which are believed to exist in all cellular compartments of oxidative metabolizing cells. The CSDs are mainly distributed in the cytosol, chloroplasts, and peroxisomes, making them the most widely distributed and largest family. Fe-SODs are mainly located in chloroplasts and are responsible for the detoxification of ROS produced during photosynthesis. Mn-SODs are mainly distributed in the mitochondria and are responsible for the scavenging of ROS produced during respiration [15,16]. Eight members of the rice SOD gene family have been reported, including four CSDs, two Fe-SODs, one Mn-SOD, and one copper chaperone protein [17].

SOD family genes play a crucial role in responding to diverse abiotic stresses by scavenging reactive oxygen species (ROS) generated under these conditions, thus contributing to the maintenance of cellular metabolism. In ginger (*Zingiber officinale* Roscoe) plantlets, the activity of SOD exhibits a gradual increase with prolonged exposure to high temperature and intense light. Together with other phenotypes, they hypothesize that ginger plants could activate multiple stress response pathways, including SOD and CAT antioxidant defenses, under high temperature and intense light conditions [18]. In the case of rice, plants overexpressing *OsCSD* exhibit enhanced germination percentage, survival rate, and plant biomass under salt stress. This suggests that OsCSD possesses the capability to mitigate salt-induced oxidative damage [19]. Environmental factors, such as temperature and humidity, strongly influence seed aging in rice. In rice, artificial aging (the seeds were treated with high temperature and humidity) elevated the levels of O_2_$\bar{\cdot }$ and H_2_O_2_ in seeds and accompanied by a reduction in the expression of *OsCSD* [20]. Additionally, the overexpression of *NbCSD* and *ascorbate peroxidase* (*APX*) in tobacco improves seed storability by reducing ROS accumulation in seeds after aging [21].

Despite the considerable number of investigations into SOD genes, conclusive evidence regarding their direct impact on seed storability in rice remains elusive. Therefore, in this investigation, we conducted a comprehensive screening of candidate genes utilizing a collection of 252 core rice germplasms. Employing genome-wide association analysis, we aimed to unravel the genetic basis of seed storability. Subsequently, we validated the seed storability phenotype through the examination of transgenic lines featuring candidate genes *OsCSD2* and *OsCSD3*. Additionally, our study explored the physiological mechanisms governing the regulation of seed storability.

## 2. Results

### 2.1. Identification of QTLs for Seed Storability by GWAS

To dissect the genetic variations in seed storability in rice germplasm, the germination behaviors were examined in a natural population containing 252 rice accessions (consisting of 152 *indica*, 84 *japonica* and 16 intermediate types). The germination parameters were recorded every 3 months starting from 6 months after harvest (no dormancy) until the seeds were stored for 24 months. P50, which is the time for the germination percentage to decrease to 50%, was calculated to determine the seed storability. A genome-wide association study (GWAS) was implemented with P50 using a linear mixed-model approach in GAPIT, and the results were presented in our previous research [22]. A Manhattan plot was generated, and 13 SNP associations with seed storability exceeding the threshold value of 5 × 10^−4^ were identified ([22], Figure S1). Among them, the peak SNP on chromosome 1 called *qSL1.2* was verified by fine mapping and further confirmed to be *OsGH3-2* by transgenic lines [22]. In the present study, we focused on *qSL3* at the top of chromosome 3, and *qSL7.2* and the end of chromosome 7 (Figure S1).

### 2.2. Identification of Potential Candidate Genes

**Figure 1:**
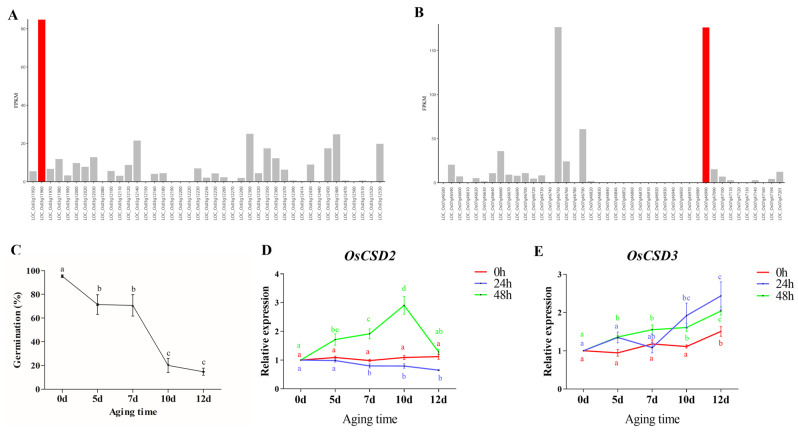
Expression analyses of the candidate genes of *qSL3* and *qSL7.2*. (**A**) The expression level of genes within the 200 kb range of the peak SNP of *qSL3* R0306454554AG in seed embryos. The red bar indicated *OsCSD2*. (**B**) The expression levels of genes within the 200 kb range of the peak SNP of *qSL7.2* F0728018966GA in seed embryos. The red bar indicated *OsCSD3* (**C**) Germination percentage after artificial aging in ZH11 seeds. (**D**,**E**) Relative expression changes of *OsCSD2* and *OsCSD3* during artificial aging and imbibed for 0, 24 and 48 h, respectively. Note: Different letters denote significant difference at *p* < 0.05 by LSD.

All of the potential candidate genes within 200 kb upstream and downstream of the leading SNP R0306454554AG for *qSL3* were analyzed. A total of 41 candidate genes were screened based on functional annotation. To further narrow down the candidate genes, we analyzed the gene expression abundance of 41 genes in seed embryos according to the RNA-seq data from the Rice Genome Annotation Project database (http://rice.uga.edu/, accessed on 21 December 2021). The results showed that the expression level of LOC_Os03g11960 was much higher than that of all the other genes (Figure 1A), which suggested that it might be the causal gene regulating seed storability in rice. The gene is named *OsCSD2*, encoding copper–zinc superoxide dismutase (Cu/Zn-SOD).

The same method was used to analyze the candidate genes of *qSL7.2*. A region 200 kb upstream and downstream of the leading SNP F0728018966GA was screened, and 2 candidate genes with higher expression in seed embryos were identified from 42 genes (Figure 1B). One candidate gene, LOC_Os07g46750, encodes an elongation factor that promotes polypeptide chain extension during mRNA translation, while the other gene, LOC_Os07g46990, encodes superoxide dismutase (*OsCSD3*), which belongs to the same family as the candidate gene of *qSL3*. *OsCSD3* had a 75 kb distance from the leading SNP. Previous reports showed the responsive expression and activity changes of SOD during rice seed aging, indicating that *OsSODs* may also be involved in the regulation of seed storability [20]. Taken together, we chose LOC_Os07g46990 to be the candidate gene for *qSL7.2* regarding seed storability.

### 2.3. Expression Changes of OsCSD2 and OsCSD3 in Aged Rice Embryos

To explore whether *OsCSD2* and *OsCSD3* were involved in seed storability regulation, the expression profiles of these two genes were investigated during seed aging for 0, 5, 7, 10 and 12 d. The germination percentage of ZH11 seeds dropped from 95% to 71%, 71%, and 20% to 14%, respectively (Figure 1C), indicating that artificial aging treatment for 10 d and 12 d dramatically reduced rice seed vigor. Although the expression profile of *OsCSD2* showed no variation in dry seeds and imbibed for 24 h, the expression of *OsCSD2* increased with aging time, peaked on the 10th day of aging, and then decreased on the 12th day (Figure 1D), and the expression of *OsCSD3* increased with aging time in dry seeds and seeds imbibed for 24 and 48 h (Figure 1E). These results indicated that *OsCSD2* and *OsCSD3* responded to the damage caused by seed aging stress.

### 2.4. OsCSD2 and OsCSD3 Positively Regulated Seed Storability

**Figure 2:**
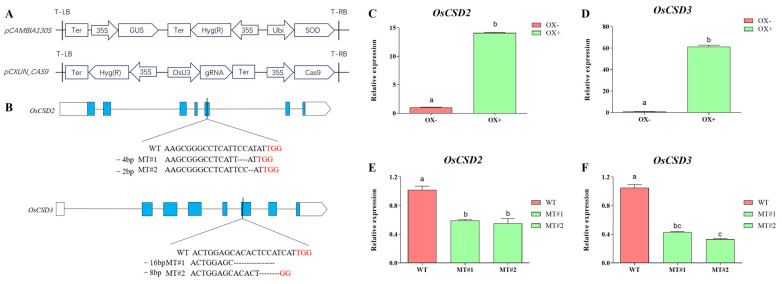
Construction strategy of *OsCSD2* and *OsCSD3* overexpression and knockout lines. (**A**) Schematic diagram of the overexpression vector (pCAMBIA130S) and CRISPR/Cas9 mutant vector (pCXUN_CAS9). (**B**) The target site editing situation of the CRISPR/Cas9 mutants. (**C**–**F**) The relative expression level of the transgenic lines. OX− represents negative control of the overexpression line (the negative plant that did not contain the transgenic element), OX+ represents the positive overexpression line, WT, wild-type plants, MT#1 and MT#2 represents the mutant line generated by CRISPR/Cas9. Note: Different letters denote significant difference at *p* < 0.05 by LSD.

To further confirm the involvement of *OsCSD2* and *OsCSD3* in seed storability, overexpression lines and CRISPR/Cas9 mutants were generated for *OsCSD2* and *OsCSD3*, respectively, in the variety ZH11 (Figure 2A,B). The expression profiles showed that the overexpression of *OsCSD2* resulted in almost 15 times higher expression, while *OsCSD3* had 60 times higher expression (Figure 2C,D). Gene disruption by CRISPR/Cas9 resulted in significantly reduced expression of *OsCSD2* and *OsCSD3* (Figure 2E,F).

**Figure 3:**
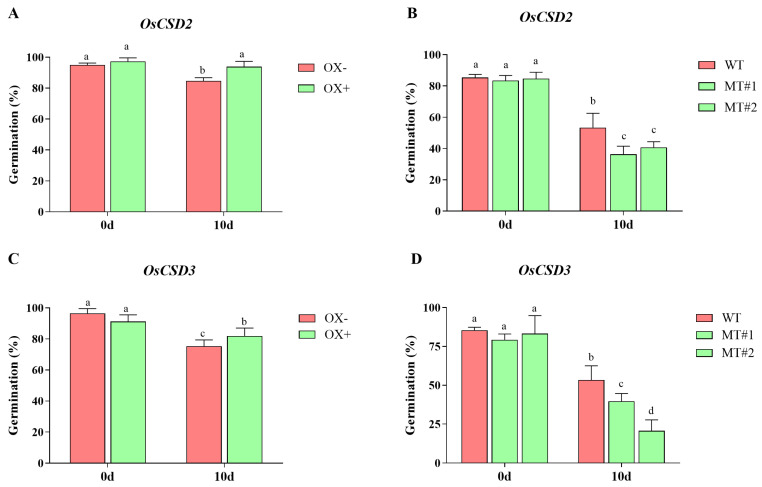
Storability of *OsCSD2* and *OsCSD3* transgenic lines. (**A**) Germination percentage of *OsCSD2* overexpression line. (**B**) Germination percentage of *OsCSD2* mutants. (**C**) Germination percentage of *OsCSD3* overexpression line. (**D**) Germination percentage of *OsCSD3* mutants. Note: The x-axis 0 d and 10 d indicate seeds without aging (0 d) and after aging (10 d), respectively. OX− represents negative control of the overexpression line (the negative plant that did not contain the transgenic element), OX+ represents the positive overexpression line, WT, wild-type plants, MT#1 and MT#2 represents the mutant line generated by CRISPR/Cas9. Note: Different letters denote significant difference at *p* < 0.05 by LSD.

Next, we analyzed the germination percentage after artificial aging for the transgenic lines of *OsCSD2* and *OsCSD3*. For *OsCSD2*, the overexpression line showed a significantly higher germination percentage after artificial aging compared with the control (Figure 3A), while the disruption of *OsCSD2* resulted in reduced seed storability compared with the corresponding WT (Figure 3B). *OsCSD3* had the same trends as *OsCSD2*. The overexpression line of *OsCSD3* was more storable than the control (Figure 3C), while the *OsCSD3* mutants had significantly reduced storability (Figure 3D). Therefore, we confirmed that *OsCSD2* and *OsCSD3* played an important role in regulating rice seed storability.

### 2.5. Phylogenetic Tree, Putative Promoters and Gene Structure Analysis of OsCSD2 and OsCSD3

**Figure 4:**
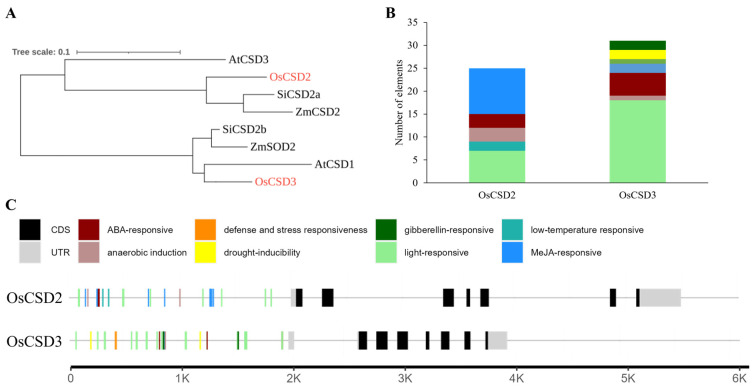
Phylogenetic tree and analysis of *cis*-acting elements and their distribution in *OsCSD2* and *OsCSD3*. (**A**) Phylogenetic tree of *CSD2* and *CSD3* in *Oryza sativa*, *Arabidopsis thaliana*, *Zea mays* and *Setaria italica*. (**B**,**C**) Number and distribution of *cis*-acting elements in the promoters of *OsCSD2* and *OsCSD3*, respectively. Note: The color in B and C represent the same motif.

To investigate the function of *OsCSD2* and *OsCSD3*, we performed a phylogenetic analysis with orthologs from other related species, such as Arabidopsis (*Arabidipsis thaliana*), maize (*Zea mays*) and millet (*Setaria italica*). *OsCSD2* was closely related to *SiCSD2a* in millet, and *OsCSD3* was closely related to *AtCSD1* in Arabidopsis (Figure 4A).

To further understand the responsive elements in *OsCSD2* and *OsCSD3*, we performed *cis*-acting element analysis in their promoters and found that 8 types of *cis*-acting elements were widely distributed in the promoter regions of *OsCSD2* and *OsCSD3* (Figure 4B,C). Light-responsive *cis*-elements accounted for the majority of elements in the promoters of *OsCSD2* and *OsCSD3*, which indicated that light stimulation can regulate their transcription. Moreover, some stress- and hormone-responsive *cis*-elements were identified in the promoter regions, including drought, low temperature, anaerobic and other defense-related elements and hormone-inducing elements such as ABA, GA and MeJA. This indicated that *OsCSD2* and *OsCSD3* may regulate the seed aging response through hormone adjustment.

### 2.6. Expression Patterns and Subcellular Localization of OsCSD2 and OsCSD3

**Figure 5:**
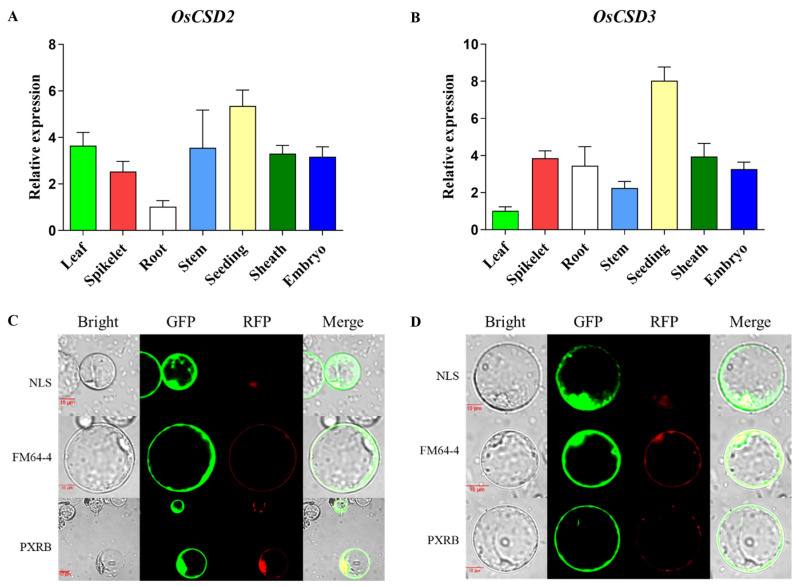
*OsCSD2* and *OsCSD3* expression profiles in different tissues and subcellular localization. (**A**,**B**) *OsCSD2* and *OsCSD3* expression profiles in different tissues. (**C**,**D**) Subcellular localization of OsCSD2 and OsCSD3. NLS, FM64-4 and PXRB are markers for nuclear, membrane and peroxisome, respectively. The red bar indicates 10 µm.

To explore the function of *OsCSD2* and *OsCSD3* during rice growth and development, we employed qRT–PCR to analyze their tissue expression profiles (Figure 5A,B). The results showed that *OsCSD2* and *OsCSD3* were expressed in the tested tissues and did not have strong specificity. They both had relatively high expression in seedlings, and there was moderate expression in the embryo. This result indicated that these two genes play an important role in the process of seed formation and seedling formation.

Next, to study which organelles OsCSD2 and OsCSD3 play a role in scavenging ROS, we performed subcellular localization analysis (Figure 5C,D). The results showed that OsCSD2 and OsCSD3 localize in the nucleus, cytoplasm and cell membrane and overlap well with the peroxisome marker PXRB (RFP).

### 2.7. Physiological Basis of OsCSD2 and OsCSD3 in Regulating Seed Storability

**Figure 6:**
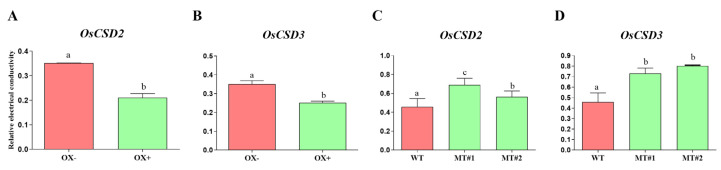
Relative electrical conductivity of *OsCSD2* and *OsCSD3* transgenic lines after artificial aging. (**A**,**B**) The relative electrical conductivity of the *OsCSD2-* and *OsCSD3*- overexpression lines. (**C**,**D**) Relative electrical conductivity of the *OsCSD2-* and *OsCSD3*- knockout mutants. OX− represents negative control of the overexpression line (the negative plant that did not contain the transgenic element), OX+ represents the positive overexpression line, WT, wild-type plants, MT#1 and MT#2 represents the mutant line generated by CRISPR/Cas9. Note: Different letters denote significant difference at *p* < 0.05 by LSD.

During the artificial aging process, high temperature and humidity aggravate the peroxidation of the cell membrane and cause electrolyte leakage. Hence, the electrical conductivity can reflect the degree of damage to the cell membrane. The relative conductivity after artificial aging was determined for the overexpression lines and the mutant lines of *OsCSD2* and *OsCSD3*. For both genes, the relative conductivity of their overexpression lines was significantly lower than that of the control, while the relative conductivity of the mutants was significantly higher than that of the wild type (Figure 6). The results indicated that the cell membrane damage of the overexpression line was less and that of the mutants was greater.

**Figure 7:**
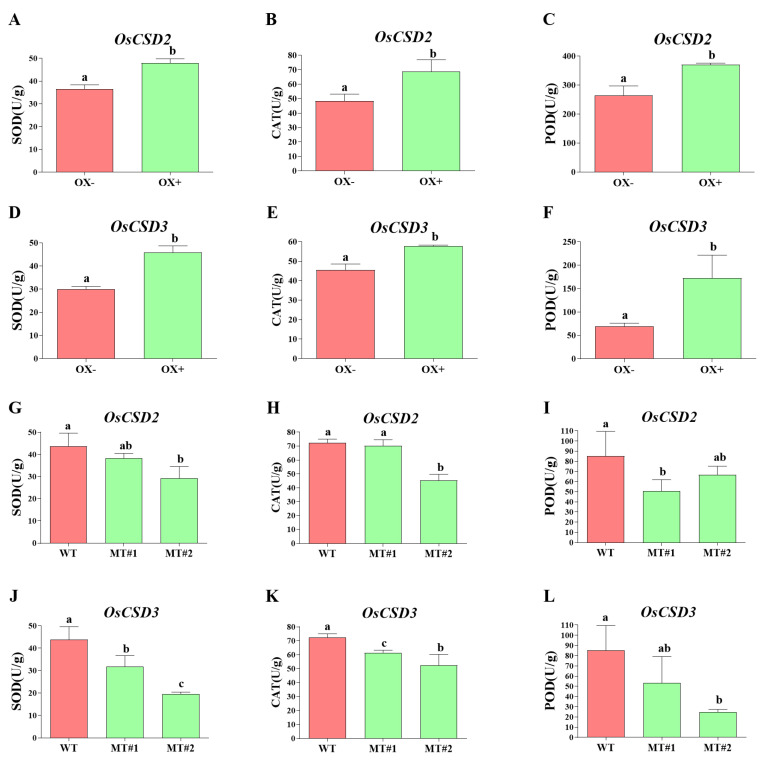
Eenzyme activities of SOD, CAT and POD in *OsCSD2* and *OsCSD3* transgenic lines after artificial aging. (**A**–**C**) SOD, CAT and POD activity of the *OsCSD2* overexpression line. (**D**–**F**) SOD, CAT and POD activity of the *OsCSD3* overexpression line. (**G**–**I**) SOD, CAT and POD activity of *OsCSD2* mutant lines, respectively. (**J**–**L**) SOD, CAT and POD activity of *OsCSD3* mutant lines. OX− represents negative control of the overexpression line (the negative plant that did not contain the transgenic element), OX+ represents the positive overexpression line, WT, wild-type plants, MT#1 and MT#2 represents the mutant line generated by CRISPR/Cas9. Note: Different letters denote significant difference at *p* < 0.05 by LSD.

To determine whether the changes in the superoxide dismutase activity of the transgenic lines affected the activities of other downstream key enzymes in the antioxidant system and ultimately caused differences in germination percentage after aging. The activities of SOD, CAT and POD in the transgenic seeds after artificial aging were measured. As expected, in the overexpression lines of *OsCSD2* and *OsCSD3*, the enzyme activity of SOD significantly increased compared with the control, and the enzyme activities of CAT and POD also increased correspondingly, reaching a significant level compared with the control (Figure 7A–F). In the mutants of *OsCSD2*, only MT#2 had significantly lower SOD and CAT enzyme activities than the wild type, while that of MT#1 was lower than that of the WT but did not reach the significance level (Figure 7G,H). The enzyme activity of POD of both mutants was lower than that of the wild type but not at a significant level (Figure 7I). In the mutants of *OsCSD3*, SOD and CAT enzyme activities of both mutants were significantly lower than wild type (Figure 7J,K), but only MT#2 had significantly lower POD enzyme activity compared with that of the wild type (Figure 7L). This indicated that overexpression or knockout of *OsCSD2* or *OsCSD3* can change SOD enzyme activity and affect the activity of downstream antioxidant enzymes in aged seeds. The changes in the activity of these enzymes resulted in differences in the germination percentage of seeds after artificial aging.

**Figure 8:**
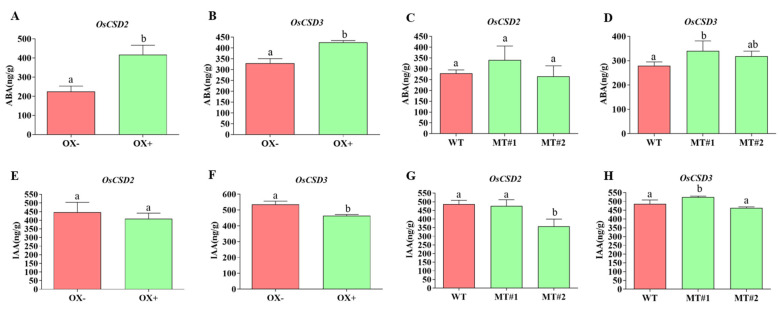
ABA and IAA contents of *OsCSD2* and *OsCSD3* transgenic seeds after artificial aging. (**A**,**B**) ABA content of the *OsCSD2-* and *OsCSD3*-overexpression lines. (**C**,**D**) ABA content of the *OsCSD2-* and *OsCSD3*-knockout mutants. (**E**,**F**) IAA content of the *OsCSD2-* and *OsCSD3*-overexpression lines. (**G**,**H**) IAA content of the *OsCSD2-* and *OsCSD3*-knockout mutants. OX− represents negative control of the overexpression line (the negative plant that did not contain the transgenic element), OX+ represents the positive overexpression line, WT, wild-type plants, MT#1 and MT#2 represent the mutant line generated by CRISPR/Cas9. Note: Different letters denote significant difference at *p* < 0.05 by LSD.

Seed germination is strongly associated with ABA levels [23], together with *cis*-element analysis in the promoters of *OsCSD2* or *OsCSD3* (Figure 4B,C). We found that the overexpression lines of both *OsCSD2* and *OsCSD3* had higher ABA contents than the wild type (Figure 8A,B), indicating that the up-regulation of *OsCSD2* or *OsCSD3* had a certain impact on the ABA metabolic pathway and probably played a certain role in prolonging seed storability. However, there was no significant change in the mutants of either gene (Figure 8C,D). For the content of IAA, there was no significant difference between the transgenic lines of both genes and the control group (Figure 8E–H), indicating that they were not related to the IAA regulatory pathway, which was consistent with the previous promoter analysis showing that there were no auxin response elements in the promoter regions of *OsCSD2* and *OsCSD3* (Figure 4B,C).

## 3. Discussion

### 3.1. OsCSD2 and OsCSD3 Are Candidate Genes for Rice Seed Storability

In the present study, 13 seed storability loci were detected by GWA analysis, located on chromosomes 1, 2, 3, 6, 7, 9, 11 and 12 (Figure S1). The candidate gene for *qSL1.2* was confirmed to be *OsGH3-2* [22] through fine mapping and transgenic line verification. *qSL9.1* contains the reported locus *qLG9* located in Nipponbare/Kasalath BILs [7], while *qSL2* is located at the top of chromosome 2, close to the reported seed storability locus *qSS2-2* detected in SN265/Luhui 99 RILs. *qSL6.3* overlaps with the *qSS6-1* detected in this study [24]. For *qSL3* and *qSL7.2*, combined with functional annotation of genes within 200 kb of the peak SNPs, as well as expression abundance analysis, it was speculated that the SOD family genes *OsCSD2* and *OsCSD3* were candidate genes within those two loci.

In our research, the germination percentage of *OsCSD2* and *OsCSD3* overexpression and knockout lines after artificial aging was analyzed, and the overexpressing lines of *OsCSD2* or *OsCSD3* had significantly higher seed storability than the controls, while the knockout lines of *OsCSD2* or *OsCSD3* had significantly lower seed storability than the wild-type seeds (Figure 3). Therefore, we confirmed that *OsCSD2* and *OsCSD3* positively regulate the seed storability of rice. *OsCSD2* and *OsCSD3* are both genes encoding copper–zinc superoxide dismutase, members of the rice SOD gene family and the first line of defense in the antioxidant system. They disproportionately use O_2_^−^ to generate O_2_ and H_2_O_2_, which are at the core of the antioxidant system [25]. To our knowledge, this is the first study to confirm that SOD family genes *OsCSD2* and *OsCSD3* participate in seed storability regulation by transgenic lines.

### 3.2. Enhancing the Antioxidant System Is Important for Prolonged Seed Storability

It has long been assumed that oxidative stress is the key factor that deteriorates seed viability during storage and that ROS accumulation is detrimental to seed viability [26]. The large accumulation of ROS in seeds can cause different levels of damage, such as peroxidation of unsaturated fatty acids in lipid membranes, abnormal protein structure and activity, and DNA single- and double-strand breaks, resulting in reduced seed storage tolerance [27,28]. Seed antioxidant enzymes are important enzymatic systems regulating seed storability. Cysteine peroxidase (1-cys peroxiredoxin, PER1) is a seed-specific antioxidant enzyme that protects cysteine and DNA from ROS. Lotus *NnPER1* is induced by stress conditions, and overexpression of *NnPER1* in Arabidopsis can enhance plant ROS scavenging ability and enhance seed vigor [29]. Recent studies have shown that the activation of *PER1* in rice seeds is regulated by two bZIP transcription factors (bZIP23 and bZIP42), which can improve seed vigor by scavenging ROS in seeds [30].

Several studies have shown that the SOD gene family is widely involved in the plant stress response. Overexpression of *Cu/Zn-SOD* and *APX* in tobacco significantly reduces ROS accumulation, reduces cell membrane damage after aging and improves seed storability [21]. The *apx6* mutant of *Arabidopsis thaliana* accumulates a large amount of ROS, leading to oxidative damage, and seed storability and seed vigor decrease under stress conditions such as oxidation and high temperature. The scavenging of ROS involves a series of enzymatic reactions and cellular metabolism. Increasing the activity of a single enzyme alone does not significantly affect the antioxidant capacity, and only the synergistic action of multiple antioxidant enzymes can promote the effective scavenging of ROS. Transforming tobacco with petunia chloroplast Cu/Zn SOD increased its SOD activity by 30 times, but its tolerance to oxidative stress caused by herbicides did not increase [31], as the activity of catalase or peroxidase did not increase accordingly, leading to intracellular H_2_O_2_ accumulation, which inhibited SOD enzyme activity on one hand and easily formed more stable and active OH^−^ on the other hand [32].

We measured the SOD, CAT and POD enzyme activities of *OsCSD2* and *OsCSD3* transgenic lines after artificial aging and observed that the three enzyme activities of the overexpressed lines were significantly higher than those of the control, while the three enzyme activities of the knockout lines were lower than those of WT, with the POD enzyme activity showing a smaller difference (Figure 7). Thus, our results indicated that *OsCSD2* and *OsCSD3* can promote an increase in downstream CAT and POD activities under aging stress, synergistically improving seed storability. In addition, the modest alternations of the knockout lines could be caused by the functional redundancy of other SOD family genes, in which a similar phenomenon was observed by Yin, Xin, Song, Chen, Zhang, Wu, Li, Liu and Lu [20]. They analyzed the expression levels of different *SOD* genes after artificial aging in rice and showed that *Cu/Zn-SOD* expression levels were decreased, *Mn-SOD* had no significant changes, while *Fe-SOD* displayed a trend of increase and then decrease, ultimately resulting in no significant changes in SOD enzyme activity during aging [20].

### 3.3. ABA Regulates Seed Storability in Relation to the Antioxidant System

ABA is known to play an essential role in the acquisition of seed longevity [33,34,35]. Numerous studies have concluded that decreased seed longevity in ABA biosynthesis and signaling mutants is due to defective ABA-mediated accumulation of seed reserves (such as storage proteins and raffinose family oligosaccharides) necessary for survival over the storage period [36,37]. In the study of ABA regulation of seed storability, it was found that *ABI3* played a central role in regulating seed storability. The ABA-insensitive mutant *ABI3* showed reduced seed storability [23]. Although the role of ABA in seed storability is supported by numerous studies, the implications of other hormones are less well documented. In addition to the interactions of HaHSFA9 and the Aux/IAA protein HaIAA27 described [38], longevity genes in *M. truncatula* were found to be enriched in binding sites for auxin-binding factors [37], implying that auxin may have a role in seed storability. Yuan, Fan, Wang, Tian, Zhang, Sun, He and Yu [22] used natural populations and RILs of Nipponbare/9311 to jointly detect a seed storability QTL on chromosome 1 and mapped it to *GH3-2* through positional cloning. *OsGH3-2* has the catalytic activity of IAA amination and reduces the free IAA content. Overexpression of *OsGH3-2* resulted in a significant reduction in seed storability and free IAA content, indicating that IAA may positively regulate seed storability.

However, few studies have provided evidence on the role of ABA in regulating seed storability in relation to the antioxidant system. Bizouerne, et al. [39] used temporal RNA-seq analyses of the different seed tissues during tomato seed maturation and constructed gene co-expression networks associated with seed storability. In addition to genes involved in protection, such as LEA and HSP and ABA response, the module also included antioxidant genes, such as 1-cys-peroxiredoxins (PER1), which protects cysteine and DNA from ROS and was proven to enhance seed storability by increasing plant ROS scavenging ability [29].

In our study, by measuring the ABA and IAA contents of transgenic materials of *OsCSD2* and *OsCSD3* after artificial aging, the ABA content in the overexpression lines was significantly higher than that in the negative control, while the ABA content in the knockout lines showed no significant change (Figure 8). In addition, there are a certain number of ABA response *cis*-acting elements distributed in the promoter regions of *OsCSD2* and *OsCSD3* (Figure 4B,C), indicating that the up-regulation of the two SOD genes has a certain impact on ABA-mediated regulation of seed longevity. The lack of effect on the knockout lines may be due to functional redundancy in the SOD family genes. In addition, there was no significant change in IAA content (Figure 8E–H), which is also consistent with the analysis results of no IAA response *cis*-acting elements in the promoter regions of *OsCSD2* and *OsCSD3*, indicating that changing the antioxidant levels of the two SOD genes would not affect the IAA regulatory pathway.

## 4. Materials and Methods

### 4.1. Plant Material

The 252 natural population materials used in the association analysis in this study for seed storability assessment [40,41] included 152 indica rice varieties, 82 japonica rice varieties and 16 intermediate rice varieties. Their genotypes were analyzed using the RiceSNP50 array as described previously [42].

### 4.2. Seed Storability Assessment

To assess the seed storability of rice germplasm, a germination test on 252 rice accessions was performed after storage for 0, 6, 9, 12, 15, 18, 21 and 24 months under stable storage conditions (25 °C; relative humidity RH, 65%) [22]. From the survival curves of germination at the indicated time points from 6–24 months of storage, seed storability represented by P50 was determined using the sigmoidal equation with GERMANATOR package [43].

To verify the storability of transgenic seeds, we first equilibrated harvested seeds in a storage cabinet (HZD-1600II, Biofuture (Beijing) Technology Co. Ltd., Beijing, China) with low relative humidity (RH 12%) for one month to obtain a constant moisture content of approximately 12% and relieve seed dormancy at the same time. Seed deterioration occurred during this process under the above conditions with 44 °C and 95% RH, termed artificial aging, which can accelerate seed deterioration and shorten the experimental process.

The germination experiments were performed in Petri dishes with three filter papers containing 15 mL distilled water and germinated at 25 °C for 7 days. All germination experiments were repeated three times for each sample, with 50 seeds per replicate.

### 4.3. Genome-Wide Association Analysis

The hybrid linear model (Q+K) in GAPIT software [44] (https://zzlab.net/GAPIT/ accessed on 13 April 2020) was used to perform genome-wide association analysis to determine SNPs that are significantly associated with seed storability using the rice core germplasm 60 K chip genotype and seed storability data on the R-studio platform (https://www.rstudio.com/, accessed on 13 April 2020). The significance threshold of the whole genome was set as a *p* value = 5 × 10^−4^; that is, the LOD was greater than 3.3. The positions of SNPs were referenced to the rice genome assembly MSU7 (http://rice.plantbiology.msu.edu/, accessed on 22 December 2022).

### 4.4. Vector Construction and Rice Transformation

To construct the overexpression vector, the *OsCSD2* and *OsCSD3* coding regions from ZS97 were cloned and inserted into linearized pCAMBIA1301s driven by the CaMV 35S promoter [45]. The CRISPR/Cas9 vectors were constructed following a previously described method [46]. All constructs were confirmed by sequencing, introduced into *Agrobacterium tumefaciens* strain EHA105, and transferred into the rice variety ZH11. All primers used for the transgenic experiments are listed in the Supplemental Information, Table S1.

### 4.5. qRT–PCR Analysis

To analyze the response expression of *OsCSD2* and *OsCSD3*, embryos (70 mg) were extracted from 0-, 24- and 48 h imbibed rice seeds that were artificially aged for 0, 5, 7, 10 and 12 d, and the samples were ground to a fine powder under liquid nitrogen. All samples were analyzed using three biological replicates, total RNA was isolated using the RNAprep Pure Plant Plus Kit (TIANGEN, Beijing, China), and cDNA synthesis was carried out using FastKing gDNA Dispelling RT SuperMix (TIANGEN, China).

Root, stem, leaf, sheath, spikelet, seedling and embryo from ZH11 were used for the tissue expression profile analysis. Fresh tissues were directly frozen in liquid nitrogen and ground to a fine powder. Total RNA from all tissue samples was extracted using the TransZol Kit (TransGen Biotech, Beijing, China). The RNA was treated with DNase I and used for first-strand cDNA synthesis with MLV reverse transcriptase (Invitrogen, Carlsbad, CA, USA).

Transcript levels were assayed using SYBR Green Master (Roche Diagnostics, Mannheim, Germany) with the ABI 7500 Real-time PCR System, and 10 μL of the reaction mixture was added to each well. Experiments were performed on three independent biological replicates and four technical replicates. Rice *UBIQUITIN* (*UBQ*) was used as the internal control, and the expression levels of the assayed genes relative to *UBQ* were analyzed using the 2^−ΔΔCT^ method, as previously described [47].

### 4.6. Subcellular Localization

The full-length CDS of *OsCSD2* or *OsCSD3* (excluding the stop codon) was inserted into a pCAMBIA1301s vector through the KpnI restriction site and then fused to the N-terminus of GFP. The recombinant constructs *CaMV 35S: OsCSD2/OsCSD3:GFP* was co-transformed into rice protoplast cells with the nuclear marker NLS-mRFP, the peroxisome marker PXRB [48], and the plasma membrane dye FM^®^ 4-64 (Invitrogen, Carlsbad, CA, USA). Rice protoplasts from seedlings of ZS97 were prepared and transformed based on the Rice Protoplast Preparation and Transformation Kit (Coolaber, Beijing, China). The fluorescent signal was observed with confocal microscopy (FV1200, Olympus, Japan) after incubating the transformed cells at 25 °C in the dark for 16 h.

### 4.7. Promoter cis-Element Analysis

The protein sequences of *OsCSD2* and *OsCSD3* and their orthologs from *A. thaliana*, *Zea mays* and *Setaria italica* in the KEGG ORTHOLOGY database (https://www.kegg.jp/kegg/ko.html, accessed on 14 September 2023) were used to construct a phylogenetic tree using MEGA5 [49] and the neighbor-joining method (p-distance model, 1000 iterations). Interactive Tree of Life (iTol) [50] (http://itol.embl.de, accessed on 14 September 2023) was used to make minor modifications to the phylogenetic tree.

The 2000 bp sequences immediately upstream from the translation start site of each gene as well as the sequence of the coding region were downloaded from the Rice Genome Annotation Project (http://rice.uga.edu/, accessed on 14 September 2023) and analyzed through PlantCARE (http://bioinformatics.psb.ugent.be/webtools/plantcare/html/, accessed on 14 September 2023). Finally, the *cis*-elements and gene structure were visualized through RStudio.

### 4.8. Relative Electrical Conductivity Measurement

The determination of seed relative conductivity was slightly modified with reference to Matthews, et al. [51]. Approximately 10 grains of full and complete brown rice were washed with ddH_2_O and dried. After transfer to a 15 mL centrifuge tube, 8 mL ddH_2_O was added, and the soaked samples were kept at room temperature for 24 h and then shaken at 120 rpm. The conductivity of the extract was measured with a conductivity meter (FE30, METTLER TOLED, USA). Then, it was heated in a boiling water bath for 30 min. After cooling to room temperature (slowly shaking on a shaker during the period), the conductivity of the extract (R2) was determined again, and the relative conductivity was calculated as R1/R2.

### 4.9. Quantification of Antioxidant Enzyme Activities

Antioxidant enzymes such as SOD, POD and CAT were extracted from seed embryos of rice imbibed for 48 h after aging for 10 days. All extraction procedures were carried out at 4 °C. Measurements were performed as follows:

SOD activity was assayed following the instructions of a SOD activity detection kit (Catalog: AKFAO001M, Boxbio, Beijing, China) by detecting the absorbance at 560 nm, and POD activity was assayed following the instructions of a POD activity detection kit (Catalog: AKFAO005M, Boxbio, China) by detecting the absorbance at 470 nm. CAT activity was assayed following the instructions of a CAT activity detection kit (Catalog: AKFAO003-2M, Boxbio, China) by detecting the absorbance at 405 nm.

### 4.10. Determination of ABA and IAA Contents

Plant endogenous phytohormones (ABA and IAA) were determined using reverse-phase liquid chromatography–tandem mass spectrometry with multiple reaction monitoring [52]. Briefly, 100 mg seeds (for each replicate) aged for 10 days were frozen in liquid nitrogen, ground to a fine powder, and extracted with 2 mL buffer containing 5 ng mL^−1^ internal standards ABA-D6 and IAA-D2. This process was carried out under dark conditions. Triple quadrupole liquid mass spectrometry was used for determination, and the relative signal intensities of endogenous phytohormones were normalized by first dividing them by the intensities of the internal standard and then log2 transformation to improve normality. Three biological replicates were measured for phytohormone determination.

## 5. Conclusions

In addressing the pressing challenge of seed deterioration during storage in rice production, this study focused on investigating candidate genes associated with identified QTLs for seed storability. Notably, the examination of two *SOD* genes, *OsCSD2* and *OsCSD3*, through overexpression and CRISPR/Cas9 mutant transgenic lines revealed their positive regulatory role in enhancing rice seed storability. Physiological investigations showed that overexpressed lines had less cell membrane damage. Additionally, overexpressed lines displayed higher antioxidant activities, suggesting a strengthened antioxidant system in the transgenic seeds. The contribution of ABA to improved seed storability in these lines was also highlighted. Overall, these findings offer valuable insights into the regulatory mechanisms of *OsCSDs* in rice storability, holding potential applications for mitigating grain loss and enhancing global food security.

## Data Availability

All data supporting this research result can be obtained in the article and within its Supplementary Materials published online.

## Supplementary Materials

The following supporting information can be downloaded at: https://www.mdpi.com/article/10.3390/plants13020310/s1, Figure S1: Manhattan plots of genome-wide association mapping for seed storability P50. The blue horizontal line indicates a threshold value of 5 × 10^−4^. Table S1: primers used in this study.
