# Supplementary material for: OsCSD2 and OsCSD3 Enhance Seed Storability by Modulating Antioxidant Enzymes and Abscisic Acid in Rice
Source: Plants (Basel). 2024 Jan 20;13(2):310. doi: 10.3390/plants13020310 (PMC10818270; doi:10.3390/plants13020310)
Supplement: Supplementary file 1 [file plants-13-00310-s001.zip › plants-2829486-supplementary.pdf]

Supporting Information

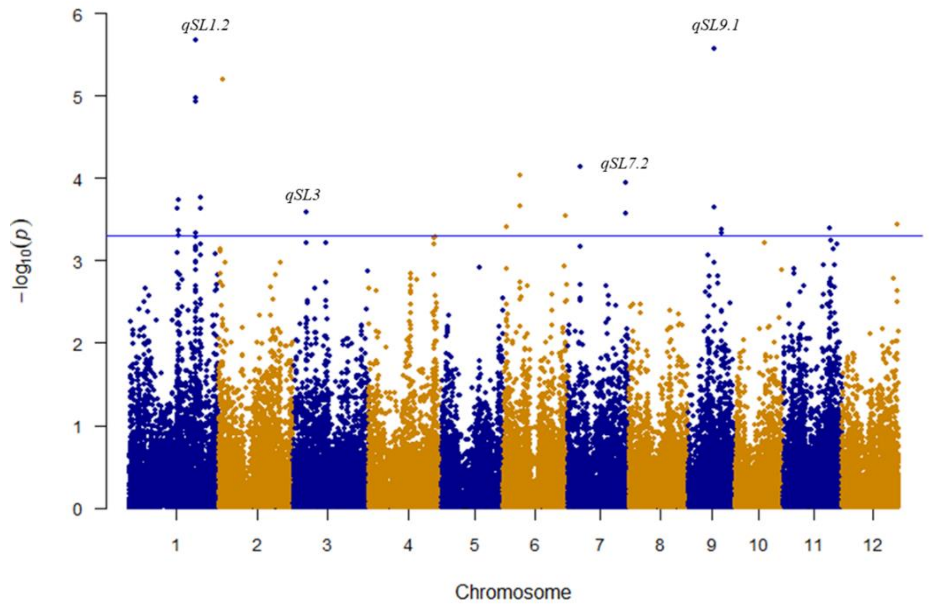

Figure S1 Manhattan plots of genome-wide association mapping for seed storability P50. The blue horizontal line indicates threshold value of $5*10^{-4}$.

Table S1 Primers used in this study.

Primer name	Sequence (from 5' to 3')	Purpose
OsU3-F	CCCCTTTCGCCAGGGGTACCgtaattcatccaggtctccaag	Vector
OsU3-R	TACGAATTCGAGCTCGGTACCgctgtgccgtacgacggtacg	construction
03g11960-cas9F	AAGCGGGCCTCATTCCATATgttttagagctagaaatagcaagtta	
03g11960-cas9R	ATATGGAATGAGGCCCGCTTgccacggatcatctgcacaactc	
07g46990-cas9F	ACTGGAGCACACTCCATCATgttttagagctagaaatagcaagtta	
07g46990-cas9R	ATGATGGAGTGTGCTCCAGTgccacggatcatctgcacaactc	
03g11960-1301s-F	atgatgatgataaaggtaccATGGCAGGGAAAGCCGG	
03g11960-1301s-R	ctagaggatccccgggtaccAACTGCAGATCGAAGTCCAATGATA	
07g46990-1301s-F	atgatgatgataaaggtaccATGGTGAAGGCTGTTGCTGTG	
07g46990-1301s-R	ctagaggatccccgggtaccACCCTGGAGTCCGATGATTC	
Cas9-F	GCATGAAGAGGATCGAGGAG	Transgenic plants
Cas9-R	GATCTCTTGCTCGGACTTGG	genotyping
03g11960-cas-F	TAATCCTCACAATAAGTCCCATG	
03g11960-cas-R	ATATCCCAGGTTAGTTGGGCTT	
07g46990-cas-F	TAGGACCACACTTCAATCCTACT	
07g46990-cas-R	AGAGAAGAGCAAGCATCATAAGA	
1301s-R	CGTTTACGTCGCCGTCCAGCT	
03g11960ox-F	TGGCTTCCACATCCACTCCT	
07g46990ox-F	CCTCTGTGACGGGAAGTGTC	
Actin-F	TGCTATGTACGTCGCCATCCAG	qPCR
Actin-R	AATGAGTAACCACGCTCCGTCA	
Ubq-qRT-F	AACCAGCTGAGGCCCAAGA	
Ubq-qRT-R	ACGATTGATTTAACCAGTCCATGA	
03g11960ox-qPCR-F	TGGCTTCCACATCCACTCCT	
03g11960ox-qPCR-R	TGGCTACTATGTTTCCCAGGTC	
07g46990ox-qPCR-F	GGCACCACAAGATGAGAACC	
07g46990ox-qPCR-R	CTCGGCCAATGATGGAGTGT	
03g11960-Rt-F	GTTCATGCTGATTCTGATGACC	
03g11960-Rt-R	GTTAAACTGCAGATCGAAGTCC	
03g22810-Rt-F	ATCCTGATGATCTTGGAAAGGG	
03g22810-Rt-R	AAAACACATAGTTCATTGGGCG	
04g48410-Rt-F	AATAAGCCTCTTGGTGACCTGGG	
04g48410-Rt-R	TCAGTGGCATACAACGCAATAG	
05g25850-Rt-F	CTACGTCGCCAACTACAACAAG	
05g25850-Rt-R	AAGATCGAATGATTGACATGGC	
06g02500-Rt-F	TCCTTCAGATGCCCTAGAGCCA	
06g02500-Rt-R	CCCACTCACTGCCACCAATC	
06g05110-Rt-F	CGAAGGTATTCAAAAGTCGTGG	
06g05110-Rt-R	AGTTCAACTGTCCTCTTGCTAA	
07g46990-Rt-F	CTTAGCAAGACCACTGGAAATG	
07g46990-Rt-R	TGTTGGAAAGTTGAGACGTCTA	
08g44770-Rt-F	AAATGCTGGTGGGCGACTTG	

08g44770-Rt-R	TGCCTCAGGCTCGAAGATGA	
03g11960-1F	CAGATTTGTCAGCGTTTGCT	Amplified full length
03g11960-1R	TGTTCCAGGTGTTGCAGA	
03g11960-2F	ATGACATAATGCTTACCCCTTC	
03g11960-2R	GTGAGAGTGGGTTTTGTTGTAC	
03g11960-3F	TGTTGGGAGAAAGTTATCGA	
03g11960-3R	GTATCTGAAAATGACCACAGTAAC	
